# Factors Influencing the Implementation of Transitional Care for Adolescents With Profound Intellectual and Multiple Disabilities: Experiences of Dutch Healthcare Professionals

**DOI:** 10.1111/jir.70101

**Published:** 2026-03-13

**Authors:** Ilse Ooms, AnneLoes van Staa, Erica Witkamp, Agnes van der Heide

**Affiliations:** ^1^ Research Centre Innovations in Care Rotterdam University of Applied Sciences Rotterdam the Netherlands; ^2^ Department of Public Health, Erasmus MC University Medical Center Rotterdam Rotterdam the Netherlands

**Keywords:** implementation, PIMD, preventing and enabling factors, profound intellectual and multiple disabilities, transitional care

## Abstract

**Background:**

The transition from paediatric to adult healthcare is particularly challenging for adolescents with profound intellectual and multiple disabilities (PIMD) and their families. This study aims to identify factors that Dutch healthcare professionals perceived as relevant to successful implementation of transitional care for adolescents with PIMD.

**Methods:**

Semi‐structured interviews were conducted with 20 professionals working in paediatric and adult healthcare. Data were analysed using directed content analysis, guided by Flottorp's checklist (2013) on preventing and enabling factors of improvements in healthcare.

**Results:**

Transitional care approaches vary at interpersonal, organisational and environmental levels. Enabling factors were parental self‐reliance and competencies, a holistic perspective, professional networks, continuity and coordination of care, flexibility to deviate from standards and guidance from nonmedical actors. Preventing factors were family burden and emotions, suboptimal information transfer, insufficient agenda‐setting, shortages of expert physicians and legal and administrative challenges. Continuous parental involvement and appropriate financial funding were enabling. The provision of person‐centred care was considered essential.

**Conclusions:**

Successful implementation of transitional care for adolescents with PIMD is a multifaceted process characterised by structural and personal challenges. Providing person‐centred care increases the likelihood of appropriate transitional care in PIMD‐care.

**Abbreviations**
AC: adult (health)care
DBC: Diagnosis Treatment Combination, a code used for financial compensation by HCPs and healthcare insurance companies
DCA: directed content analysis
GP: general practitioner
HCP: healthcare professional
ID: intellectual disability
ID Physician: intellectual disability physician
PC: paediatric (health)care
PIMD: profound intellectual and multiple disabilities
TC: transitional care

## 1 Background

In Europe and the United States, fewer than 1% of people are living with profound intellectual and multiple disabilities (PIMD) (Rousseau et al. 2024; Emerson 2009; Patel et al. 2018). PIMD is associated with severe intellectual and motor impairments (Nakken and Vlaskamp 2007); the daily functioning of afflicted individuals is largely or fully dependent on others. Healthcare professionals (HCPs) across multiple medical and paramedical disciplines are involved in their medical care (van der Putten et al. 2017; Zandbelt et al. 2025). Advances in medical care have extended the life expectancy among individuals with PIMD (Dolan et al. 2019), resulting in a growing number reaching adulthood. Upon turning 18, the adolescents need to transition from paediatric healthcare (PC) to adult healthcare (AC). This process—referred to as transitional care (TC)—is regarded as an important yet challenging turning point, often associated with great stress (Luitwieler et al. 2024; Raap et al. 2024; Geuze and Goossensen 2023). Stress induced by TC is reported across Western healthcare systems (McManus et al. 2020; Schmidt et al. 2020; Trettin et al. 2025).

TC for adolescents with PIMD is highly complex due to multimorbidity—often involving multiple organ systems (Van Timmeren et al. 2016; Mol‐Bakker et al. 2024), limited communication abilities and strong dependence on caregivers. Beyond the transfer from paediatric to adult specialists, these adolescents frequently face concurrent transitions in daycare, housing and financial regulations. Coordinated care is a key component of high‐quality healthcare for individuals with intellectual and developmental disabilities and is associated with better health outcomes (Breuer et al. 2022; Schmidt et al. 2020). Conversely, inadequate TC may lead to loss to follow‐up, personal suffering and increased societal costs (Davidse et al. 2020).

Despite its importance, research on the implementation of TC for adolescents with PIMD remains limited. Existing studies have focused either on TC in general (Gray et al. 2017; Parsons et al. 2022) or on specific interventions (Sturm et al. 2024). Reported barriers include insufficient attention to emotional needs, poor care coordination and limited preparedness of AC services (Bhaumik et al. 2011; Bindels‐de Heus et al. 2013; Brown et al. 2019). A recent European study found that family caregivers emphasised the need for personalised approaches, timely information, interprofessional communication and well‐coordinated care (Klein Haneveld, Vyshka, et al. 2025). In 2025, the first guideline specifically addressing TC for PIMD was published (Klein Haneveld, Świeczkowska et al. 2025).

Flottorp et al. (2013) identified 57 determinants across seven domains in their TICD‐checklist (The Integrated Checklist of Determinants of practice) that affect care innovation for individuals with chronic conditions. In a two‐round Delphi study conducted in 2020/2021 (not published) on the relevance of Flottorp's checklist for TC in hospitals, the expert panel considered factors in all seven domains highly relevant. To date, a comprehensive overview of HCPs' perspectives on implementation of TC is lacking. The aim of this study is to systematically explore factors influencing TC for adolescents with PIMD, as experienced by Dutch HCPs.

## 2 Methods

This explorative qualitative study used semi‐structured interviews with HCPs.

### 2.1 Participants

Twenty HCPs from various settings in the Netherlands were recruited through purposive (*n* = 10) and snowball sampling (*n* = 10) aimed at representing professionals from both PC and AC caring for adolescents or young adults with PIMD (aged 12–25 years), regional diversity, working in academic and non‐academic institutions and a range of disciplines (e.g., physicians, nurse practitioners and physician assistants).

### 2.2 Data Collection

The interviews were guided by a topic list comprising five broad themes, focusing on general information of the participants and their experience with TC, the organisation of TC, the interaction and collaboration between parents and HCPs and the factors experienced as influencing implementation (see Data S1).

**FIGURE 1:**
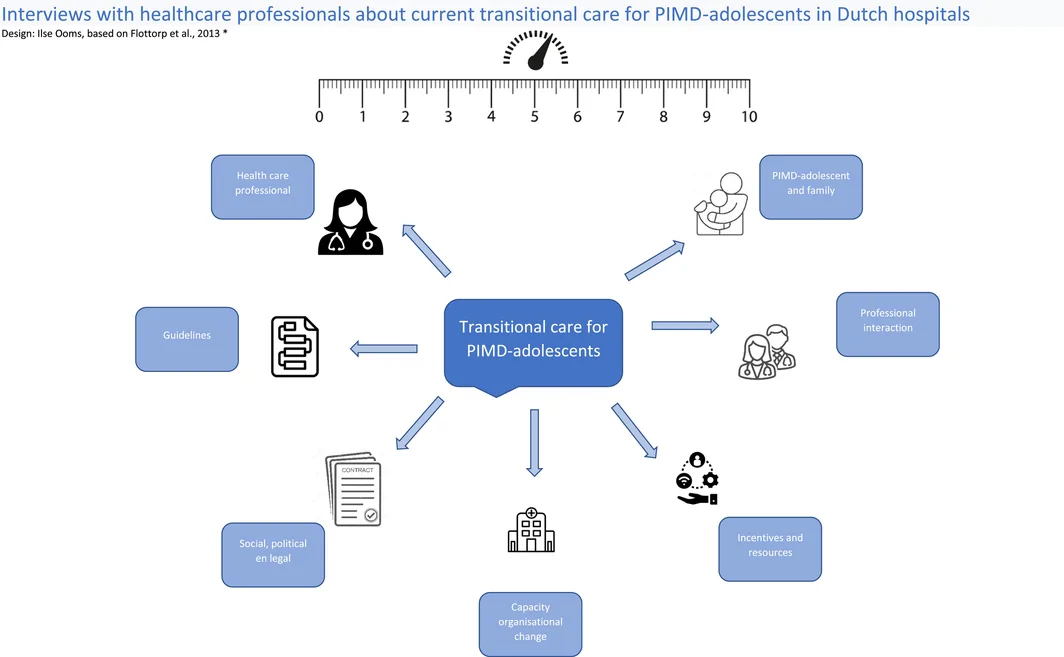
Visual used in interviews.

After participants had answered the open questions, they were shown a visual based on the seven domains of Flottorp et al. (2013) (see Figure 1) to prompt deeper reflection on factors influencing implementation of TC in their healthcare facility.

After each interview, participants received a detailed summary of their responses to verify accurate interpretation. Nineteen interviews were conducted online via MS Teams, and one was conducted in‐person. All interviews were performed by IO and audio‐recorded.

### 2.3 Data Analysis

**FIGURE 2:**
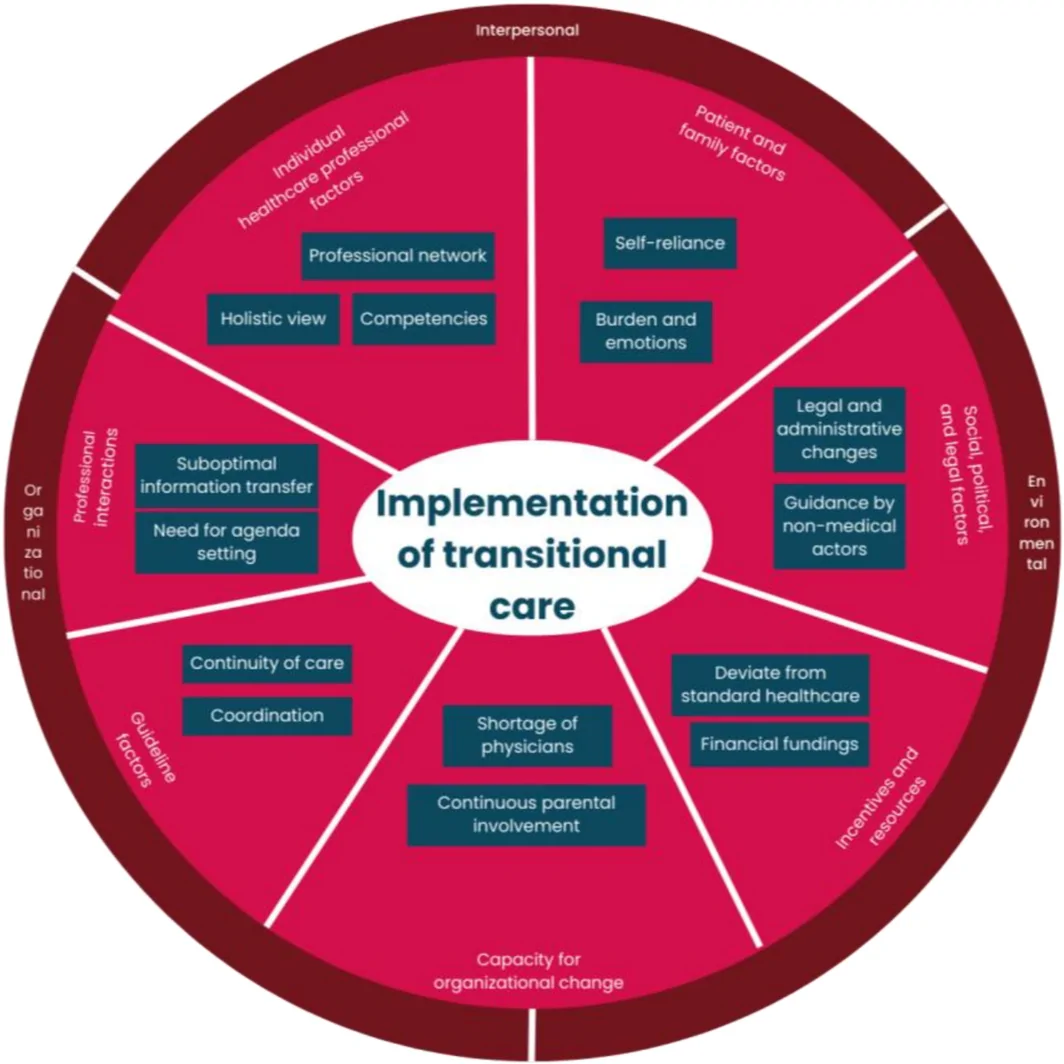
Factors influencing implementation of TC for adolescents with PIMD.

Interviews were transcribed verbatim and imported into Atlas.ti. Analysis commenced after all 20 interviews were completed. We applied the three phases of directed content analysis (DCA)—preparation, organisation and reporting—as described by Assarroudi et al. (2018). Our analysis followed four steps:

Step 1: Open coding (Data S1, left column) was combined with deductive coding, using the seven domains of Flottorp et al. (2013) as formative categories;
Step 2: Three levels of factors influencing TC implementation were found (Data S1, middle column);
Step 3: Open codes from Step 1 were mapped onto the seven domains of Flottorp et al. (2013) (Data S1, right column);
Step 4: The levels from Step 2 were paired with Flottorp domains to create an integrated framework (Figure 2).

Initial coding was performed by IO, while all authors contributed to the preparation, interpretation and analysis in Steps 2–4.

### 2.4 Ethical Approval

Written informed consent was obtained from all participants after they were provided with detailed information about the study. The Medical and Ethical Review Committee of Erasmus MC, University Medical Center Rotterdam, reviewed this study and determined that it did not fall under the scope of the Medical Research Involving Human Subject Act (MEC‐2023‐0489).

## 3 Results

Twenty HCPs were interviewed, as detailed in Table 1. After Interview 17, no new open codes were found in Interviews 18, 19 and 20.

**TABLE 1:** Characteristics of participants.

Paediatrician	*n* = 11	PC	hospital
Neurologist	*n* = 1	PC	hospital
Nurse practitioner	*n* = 1	PC	hospital
Intellectual disability physician	*n* = 5	AC	*n* = 3 ID facility *n* = 2 ID facility and hospital
Pulmonologist	*n* = 1	AC	hospital
Rehabilitation physician	*n* = 1	AC	hospital

An ID physician is a medical specialty in the Netherlands (Huisman et al. 2024).

The interviews revealed that most HCPs do not apply a protocol or guideline for TC: ‘I think the word “transition” is still quite new, isn't it? When I was trained, TC didn't exist’ (HCP13, AC).

HCPs rated the quality of their TC practices at an average of 6.2 out of 10 and highlighted the need for improvement: ‘It is a sufficient score, yes. But it is a narrow one. If it were up to me, I'd like to do so much more’ (HCP13, AC).

Factors influencing the implementation of appropriate TC were found at three levels: (inter)personal, organisational and environmental level. Combining these levels with the seven Flottorp domains led to the factors illustrated in Figure 2.

Below, we elaborate on the identified preventing and enabling factors of successful implementation:

### 3.1 Patient and Family Factors

Most HCPs asserted that PIMD is highly heterogeneous and advocated for personalised healthcare: ‘It really is a case of tailoring our approach to each individual patient: where do they come from, where do they live, what healthcare options are available in their neighbourhood, what are the main issues they face? It truly is a puzzle’ (HCP7, PC).

HCPs noted that parents experience significant administrative and logistical **burden and emotions**, next to the stress of their child's medical transfer. However, this is beyond HCPs' control:

The administrative burden on parents is very heavy; there are dozens of agencies in the Netherlands, and they all probably have the best intentions, but they also tend to work at cross‐purposes. Sometimes they even work against each other, so parents at least feel like being sent from pillar to post. (HCP2, PC)

And also in such an intense phase when often a lot happens, puberty and everything. Yes, that does a lot with a patient, but also with their family. (HCP17, AC)

According to HCPs, parents may be reluctant to have their child transferred to AC, forcing HCPs to initiate a difficult conversation about ending their close relationships with providers from PC: ‘I mainly see parents who say: “You'll still be our doctor, right?” No, I'm a paediatrician, so it's officially up to 18. [Parents wish] to maintain that familiar face’ (HCP12, PC).

Cultural and socioeconomic factors such as poverty, limited health literacy, specific cultural background, parents with an ID or a language barrier, social isolation of the adolescent due to living at home and lack of daytime activities can further limit families' access to care. This affects families' **self‐reliance**, while having to rearrange the child's life, which is considered a preventing factor:

Certainly vulnerable families. It can be difficult for parents to organise things, if you don't speak the language or lack the financial means. (HCP17 AC)

HCPs try to meet families' needs and preferences, however, AC options are limited, shared decision‐making is often not possible: ‘We have so few ID physicians and healthcare provision in general, that you should actually be happy if there is one organisation in the region that can take care of your child. So, there is not much choice, and doctors and parents generally find that quite difficult’ (HCP13, AC).

### 3.2 Individual HCP Factors

HCPs identified having suitable **competencies** as an enabling factor for implementation of TC, including expertise in hereditary and congenital conditions as well as communication skills for interacting with nonverbal people.

Adult HCPs are not at all geared towards young people with multi‐complex disorders and their behaviour – they're not trained for that. They have little experience with people with PIMD. (HCP12, PC)

A **holistic view** of health was also deemed an enabling factor, as adolescents with PIMD often live with multiple conditions and challenges in many life areas:

It's about attitude. You must dive into a patient to understand what's going on. That may take up to an hour or so. (HCP2, PC)

In adult care, at least in hospitals … everyone feels responsible for a part of a person, but nobody feels responsible for that whole person and their environment. (HCP15, PC)

HCPs stressed having a well‐established **professional network** as enabling for smooth referrals to AC specialists: ‘I have my own network, I know which neurologist is willing to see someone with a disability and which ENT doctor takes a little more time. I know who is useful to send someone with a particular problem to, so you just have your own network. That's the hardest part, building that network, and that's what you really need’ (HCP17, AC).

### 3.3 Professional Interactions

**Suboptimal information transfer** was identified as a preventing factor. HCPs highlighted inadequate referral documentation, lack of warm handovers or multidisciplinary consultations. A warm handover is a joint consultation in which the HCP from PC introduces the adolescent to the HCP from AC and officially transfers care in the presence of the adolescent and family (Cassidy et al. 2022).

On the benefit of warm handovers, HCP6 (AC) stated: ‘Well, it's nice to do it together, because you can see how the paediatrician interacts with the parents, you can see how those people interact with each other. That makes things easier. Then you just have a bit more information. And you've been introduced by a paediatrician, who says they have confidence in you’.

Interprofessional interaction is hampered when HCPs are not affiliated with the same organisation. Sharing files, requesting diagnostics and organising acute care is then more complicated. An ID physician explained:

All those stupid electronic patient files not synchronising with each other. Now, the interesting thing is that a lot of online things are not allowed. What is allowed for me is to print it and have the other [organisation] scan it. Yes, that's ridiculous! (HCP15, AC)

HCPs stressed that they need to put TC on the agenda of national politicians and policymakers (**agenda‐setting**) since organisational and environmental factors can only be resolved through a comprehensive approach:

Because, I'm also in the Dutch board of paediatricians for children with hereditary and congenital disabilities … We are the forum to take that to a higher level. What else could we do to give this topic more cachet? (HCP3, PC)

### 3.4 Guideline Factors

HCPs argued that a specific guideline for TC in PIMD could give them greater job satisfaction and be less stressful for parents: ‘I think job satisfaction would improve greatly. I see a lot of parents who are really burning out or becoming ill now, because they simply have too many worries and can no longer cope with the burden of care. So, it [a guideline] also prevents a great deal of crisis by addressing this in a very systematic manner and thoroughly screening for any potential issues’ (HCP13, AC).

Generic guidelines on TC recommend, for example, appointing a designated TC coordinator to ensure continuity of care. HCPs considered **continuity of care** the best outcome measure for appropriate TC and an enabling factor, noting that parents need a clear point of contact after their child's has transferred to AC:

If one can tell: “I know who to go to for what kind of healthcare, then I think you're well on your way.” (HCP13, AC)

HCPs elaborated on the risks of inappropriate TC. HCP11 (AC) mentioned a boy with PIMD (aged 20) who had to be admitted to and adult hospital due to pneumonia: ‘He is essentially a child. He's completely wheelchair‐dependent, and he hardly makes any contact. The good thing is, that an adult ward can provide for a slightly larger bed and perhaps a hoist for a tall person. Still, they put down a tray of food and walk away expecting that he will eat. But this boy can't do anything with that! So, an adult ward simply can't deal with these children’.

In addition, the importance of timely starting planning TC was brought up; not only to ensure continuity of care but also to prevent adolescents over the age of 18 from being seen in paediatric care without financial compensation.

HCPs shared experiences of inadequate interdisciplinary **coordination** and highlighted the absence of a designated TC coordinator in AC as a critical preventing factor.

With PIMD children, it's never just one problem, is it? It's never just the heart or just lungs, it's always multiple problems … the paediatrician oversees all those problems. And while the children see a cardiologist and a pulmonologist, the paediatrician keeps track of everything, um, the big picture. (HCP12, PC)

An ID physician stated: ‘After the transition you will set up a kind of network for, say, epilepsy, which is still very much an issue. … If you find it too complex [to treat it yourself], then make sure that they are referred to a neurologist again, acting, as it were, as a kind of director in that process’ (HCP17 AC).

### 3.5 Capacity for Organisational Change

Another factor preventing the successful implementation of TC is ***shortages of physicians***, particularly ID physicians in AC. HCPs indicated that it is already challenging to organise regular care for people with PIMD, and that the additional desired coordination and alignment is even more difficult to achieve. Shortages of ID physicians lead to waiting lists:

We [ID physicians] are uniquely capable of acting across all areas of life and we can act across all stages of life, from childhood to adulthood, so we are truly trained to oversee [TC]. And to coordinate and advise on that. Paediatricians … they would prefer to refer everyone to us. Well, that's not possible, because we only have two clinics per month. So, we would be immediately overbooked. (HCP13 AC)

AC is characterised by a high level of specialisation, where physicians focus on their own specialty. Psychiatric problems in PIMD adolescents do not always receive the necessary attention, sometimes resulting in disruptive behaviour: ‘I notice that they [co‐physicians] focus so much on transition to somatic specialists, while paying less attention to the psychosocial aspect, which means that children later develop mood problems or don't receive the right care’ (HCP13 AC).

Adolescents with PIMD need continuous parental involvement, which is often not possible in AC. HCPs emphasised the (in)ability to ensure **continuous parental involvement** post‐transfer as an influencing factor. They advocated for more specialised wards for adolescents and adults with PIMD to enable 24/7 parental presence.

Well, rooming‐in is often more difficult with us. Fortunately, it has worked so far [in our clinic]. For those who have ended up in the intensive care, uhm, a bit more impersonal, they are just a bit stricter. (HCP19, AC)

### 3.6 Incentives and Resources

The flexibility to **deviate from standard healthcare** enables HCPs to provide more personalised healthcare. They highlighted several positive examples, including extended consultations, joint consultations, TC coordinators and warm handovers. However, lack of **financial funding** remains a preventing factor:

It took me over two years to organise joint consultations [with an ID physician] here in the hospital. (HCP3, PC)

HCP4 (PC) referring to warm handovers: ‘You can only invoice a consultation once, so one person acts at their own expense. Of course, there should be some kind of transitional DBC or something else for that, but we don't even have a good DBC yet to claim the care we provide’.

DBC: Diagnosis Treatment Combination, a code used for financial compensation by HCPs and healthcare insurance companies.

Still, HCPS seemed motivated to deviate from standards and deliver healthcare not covered by insurance.

I think that in TC, in palliative care, in fact in all care for this group, there are many things you don't get paid for, but which enable you to provide good healthcare for someone. I don't think I should be paid for every minute I work. But if there were rates for certain aspects … that would put less pressure on me in terms of quantity and production. (HCP15, PC)

Some organisations are looking for creative solutions to financial barriers:

Look, if you work in a hospital with lots of separate partnerships, financing becomes very difficult. But in our hospital, everything is under one umbrella, so we are now looking into whether [name ID physician] could be employed by the umbrella organisation, so that multiple specialisms can make use of her services. But, of course, things are not organised in that way at every healthcare facility. (HCP10, PC)

### 3.7 Social, Political and Legal Factors

HCPs stated they lack the expertise or resources to guide families through nonmedical **legal and administrative** changes, such as guardianship and insurance, which lack is considered a preventing factor.

What we do explain to everyone, is how to go about things like guardianship, that sort of thing… we already alert to this when children are around the age of 16 or 17. Like, hey, it's really coming up now, remember, you also have to arrange things with the court… Sowe tell them, we do address that the legal part. (HCP1, PC)

HCPs are not trained on this topic: ‘All those regulations you have to deal with when your child turns 18, well, I didn't learn about that in college’ (HCP14, PC).

Involving other professionals, like social workers, for **nonmedical guidance by others** is deemed an enabling factor:

We used to have social workers here. That has all been cut back, so now you only have social workers for families who are really struggling, whereas in the past there was much more support. (HCP17, AC)

## 4 Discussion

This study identified a range of factors—identified by Dutch HCPs—that may influence successful implementation of TC for adolescents with PIMD. These factors were identified across all domains delineated by Flottorp et al., underscoring the need for a comprehensive approach that engages not only HCPs and families but also policymakers, healthcare and social support organisations and health insurers.

The study findings demonstrate that TC for adolescents with PIMD is a complex undertaking, which in view of the heterogeneity among this population necessitates a personalised approach. PIMD is a rare condition, and not until in recent decades have a greater proportion of adolescents reached adulthood. Consequently, (transitional) care for adolescents with PIMD is still underdeveloped. Its complexity stems from its multifaceted nature, involving multiple disciplines, and the interplay of several medical and intellectual disabilities necessitating substantial parental involvement of parents.

Frequent transfers between PC and AC, often driven by multimorbidity, further complicate information exchange and communication. Most healthcare organisations rely on non‐interconnected communication systems, and the lack of standardisation in documentation for TC increases the risk of communication errors and may lead to medical mistakes (Schmidt et al. 2020).

Adult care providers involved in this study acknowledged that they are less well equipped to address the multifaceted physical and psychosocial challenges faced by adolescents with PIMD. This phenomenon, often described as ‘siloed systems’, has also been reported by Franklin et al. (2019). Furthermore, adult HCPs are generally less accustomed to close collaboration with family members (Witkamp et al. 2016). A family‐centred approach to TC, as proposed by Kerr et al. (2020), entails the active involvement of parents and other caregivers, such as siblings or legal guardians (Kruithof et al. 2022). The present study demonstrates that healthcare organisations, regulations and funding structures remain insufficiently aligned with the needs of adolescents with PIMD.

This finding underlines the need for effective care coordination after transfer to AC. While in PC, a specialised paediatrician typically oversees complex cases, whereas in AC, there is a shortage of specialists with relevant expertise (ACMMP 2019). This gap may disrupt continuity of care and result in suboptimal health outcomes (Klein Haneveld, Vyshka, et al. 2025). Participants expressed a preference for appointing an ID physician or a skilled general practitioner as care coordinator; however, workforce shortages among these disciplines present a significant barrier.

A key finding of this study is the multifaceted parental burden during the transition period. Inadequate preparation and communication may further exacerbate this burden, consistent with the observations of Franklin et al. (2019). In their recently published PIMD TC‐guideline, Klein Haneveld, Świeczkowska et al. (2025) recommend psychosocial support and a ‘named support worker’ for planning and support. A case manager (e.g., a nurse practitioner or an independent client support worker) could offer both practical guidance and emotional support to the family. Healthcare organisations have not yet implemented the guideline recommendations.

HCPs emphasised that standard TC models are deficient for adolescents with PIMD. This observation is supported by Bert et al. (2020), who recommend an alternative clinical pathway, such as an Interdisciplinary Transition Group, for individuals with complex medical and social conditions like PIMD. Betz (2023) highlighted the need for specialised clinical and programmatic models in healthcare transition planning for individuals with intellectual or developmental disabilities. Given that individuals with PIMD may never fully integrate into standardised AC systems, transferring them to AC is therefore called into question.

## 5 Recommendations

Implementing the guideline for TC for adolescents with PIMD could assist HCPs in providing TC that meets individual and family needs. Streamlined information exchange between HCPs could reduce—potentially through a standardised transition plan—communication error (NICE 2023). Shared care models, such as those described by Culnane et al. (2023), can foster communication and collaboration between HCPs and families (Aarthun et al. 2018). Teaching internists, pulmonologists and neurologists to treat adults with PIMD can enhance care (Matérne and Holmefur 2022), minimise disruption in care and improve health outcomes (Schmidt et al. 2020). Involving parents in the transition plan would help shift the focus in AC from person‐ to family‐centred care (Kokorelias et al. 2019). Employing ID physicians within hospitals that regularly provide care to adults with PIMD may help address gaps in coordination and facilitate more direct communication. Reducing the emotional and administrative burden on parents during the transition period could further mitigate stress (Luitwieler et al. 2024). Further research on appropriate care for adults with PIMD and their families is needed.

## 6 Strengths and Limitations

Twenty HCPs were interviewed for this study, with data saturation reached after 17 interviews. Ten participants were selected based on their involvement in TC for individuals with PIMD, while an additional ten were recruited from the authors' networks. This sampling method may have introduced selection bias, however, as participants likely had a heightened interest in the subject. However, considering the small number of HCPs actively involved in TC for adolescents with PIMD in the Netherlands, the sample size is deemed reliable and highly representative across clinics, regions and medical specialties.

Assarroudi et al. (2018) argue that the trustworthiness of this type of research is heightened if the three phases and associated steps of DCA are correctly followed and reported on. It could be argued that deductive coding can lead to confirmation bias. There is only a slight likelihood of confirmation bias in this study, as the analysis used open coding; interviewees were shown a visual of known influencing factors after they had spontaneously answered all the open questions.

## 7 Conclusion

This study demonstrates that TC for adolescents with PIMD is highly complex and insufficiently supported within the current Dutch healthcare system. The heterogeneity and rarity of PIMD call for personalised, coordinated and family‐centred approaches, yet such models are not consistently implemented in practice. Barriers include fragmented communication, limited expertise in adult care, misaligned funding structures, inadequate care coordination and differing views on parental involvement. These factors increase the risk of discontinuity of care and heighten parental stress during transition. Although a recent guideline provides direction, TC delivery remains suboptimal. A comprehensive, multistakeholder approach and alternative care pathways, such as specialised PIMD services, are needed to ensure continuity and quality of care.

## Funding

This study was funded by the Dutch Research Council (NWO), grant number: 023.020.002.

## Conflicts of Interest

The authors declare no conflicts of interest.

## Supporting information


**Data S1:** Supporting information.

## Data Availability

The data that support the findings of this study are available on request from the corresponding author. The data are not publicly available due to privacy or ethical restrictions.

## References

[jir70101-bib-0001] Aarthun, A. , K. A. Øymar , and K. Akerjordet . 2018. “Parental Involvement in Decision‐Making About Their Child's Health Care at the Hospital.” Nursing Open 6, no. 1: 50–58. 10.1002/nop2.180. 30534394 PMC6279730

[jir70101-bib-0002] ACMMP, Advisory Committee on Medical Manpower Planning . 2019. “Recommendations 2021–2024, Concerning the Intake in Medical, Clinical Technological, Dental, Mental Healthcare, FZO (Hospital Training Programmes Fund), Physician Assistant, Nurse Practitioner and Related Initial Degree and Postgraduate Programmes, Utrecht, the Netherlands.” Downloaded From www.capaciteitsorgaan.nl Downloaded on September 29, 2023.

[jir70101-bib-0004] Assarroudi, A. , F. H. Nabavi , M. R. Armat , A. Ebadi , and M. Vaismoradi . 2018. “Directed Qualitative Content Analysis: The Description and Elaboration of Its Underpinning Methods and Data Analysis Process.” Journal of Research in Nursing 23, no. 1: 42–55. 10.1177/1744987117741667. 34394406 PMC7932246

[jir70101-bib-0005] Bert, F. , E. Camussi , R. Gili , et al. 2020. “Transitional Care: A New Model of Care From Young Age to Adulthood.” Health Policy 124, no. 10: 1121–1128. 10.1016/j.healthpol.2020.08.002. 32843225

[jir70101-bib-0006] Betz, C. L. 2023. “Healthcare Transition Planning for Adolescents and Emerging Adults With Intellectual Disabilities and Developmental Disabilities: Distinctions and Challenges.” Journal for Specialists in Pediatric Nursing 28, no. 3: e12415. 10.1111/jspn.12415. 37380603

[jir70101-bib-0007] Bhaumik, S. , J. Watson , M. Barrett , B. Raju , T. Burton , and J. Forte . 2011. “Transition for Teenagers With Intellectual Disability: Carers' Perspectives.” Journal of Policy and Practice in Intellectual Disabilities 8, no. 1: 53–61. 10.1111/j.1741-1130.2011.00286.x.

[jir70101-bib-0008] Bindels‐de Heus, K. G. C. B. , A. Van Staa , I. Van Vliet , F. V. P. M. Ewals , and S. R. Hilberink . 2013. “Transferring Young People With Profound Intellectual and Multiple Disabilities From Pediatric to Adult Medical Care: Parents' Experiences and Recommendations.” Intellectual and Developmental Disabilities 51, no. 3: 176–189. 10.1352/1934-9556-51.3.176. 23834214

[jir70101-bib-0009] Breuer, M. E. J. , E. B. Gijssel , K. V. Anrooij , H. Tobi , G. L. Leusink , and J. Naaldenberg . 2022. “Exploring Views on Medical Care for People With Intellectual Disabilities: An International Concept Mapping Study.” International Journal for Equity in Health 21, no. 1: 101. 10.1186/s12939-022-01700-w. 35854317 PMC9295354

[jir70101-bib-0010] Brown, M. , A. Higgins , and J. MacArthur . 2019. “Transition From Child to Adult Health Services: A Qualitative Study of the Views and Experiences of Families of Young Adults With Intellectual Disabilities.” Journal of Clinical Nursing 29, no. 1–2: 195–207. 10.1111/jocn.15077. 31610045

[jir70101-bib-0011] Cassidy, M. , S. Doucet , A. Luke , A. Goudreau , and L. MacNeill . 2022. “Improving the Transition From Paediatric to Adult Healthcare: A Scoping Review on the Recommendations of Young Adults With Lived Experience.” BMJ Open 12, no. 12: e051314. 10.1136/bmjopen-2021-051314. PMC9806082 36572498

[jir70101-bib-0012] Culnane, E. , D. Efron , K. Williams , et al. 2023. “Carer Perspectives of a Transition to Adult Care Model for Adolescents With an Intellectual Disability and/or Autism Spectrum Disorder With Mental Health Comorbidities.” Child: Care, Health and Development 49, no. 2: 281–291. 10.1111/cch.13040. 35947107

[jir70101-bib-0013] Davidse, K. , A. Staa , K. Pellikaan , et al. 2020. “SUN‐080 We Mind Your Step: Understanding and Preventing Drop‐Out in the Transition From Paediatric to Adult Tertiary Endocrine Healthcare.” Journal of the Endocrine Society 4, no. Supplement_1: SUN‐080. 10.1210/jendso/bvaa046.1192.

[jir70101-bib-0014] Dolan, E. , J. Lane , G. Hillis , and N. Delanty . 2019. “Changing Trends in Life Expectancy in Intellectual Disability Over Time.” PubMed 112, no. 9: 1006. https://pubmed.ncbi.nlm.nih.gov/31651135. 31651135

[jir70101-bib-0015] Emerson, E. 2009. “Estimating Future Numbers of Adults With Profound Multiple Learning Disabilities in England.” Tizard Learning Disability Review 14, no. 4: 49–55. 10.1108/13595474200900040.

[jir70101-bib-0016] Flottorp, S. A. , A. D. Oxman , J. Krause , et al. 2013. “A Checklist for Identifying Determinants of Practice: A Systematic Review and Synthesis of Frameworks and Taxonomies of Factors That Prevent or Enable Improvements in Healthcare Professional Practice.” Implementation Science 8: 35. 10.1186/1748-5908-8-35. 23522377 PMC3617095

[jir70101-bib-0017] Franklin, M. S. , L. N. Beyer , S. M. Brotkin , G. R. Maslow , M. D. Pollock , and S. L. Docherty . 2019. “Health Care Transition for Adolescent and Young Adults With Intellectual Disability: Views From the Parents.” Journal of Pediatric Nursing 47: 148–158. 10.1016/j.pedn.2019.05.008. 31152999

[jir70101-bib-0018] Geuze, L. , and A. Goossensen . 2023. “Caring for Children With Profound Intellectual and Multiple Disabilities: Images and Metaphors Expressed by Dutch Parents.” Disability & Society 39: 1840–1858. 10.1080/09687599.2023.2164846.

[jir70101-bib-0019] Gray, W. N. , M. R. Schaefer , A. Resmini‐Rawlinson , and S. T. Wagoner . 2017. “Barriers to Transition From Pediatric to Adult Care: A Systematic Review.” Journal of Pediatric Psychology 43, no. 5: 488–502. 10.1093/jpepsy/jsx142. 29190360

[jir70101-bib-0020] Huisman, S. , D. Festen , and E. B. Gijssel . 2024. “Healthcare for People With Intellectual Disabilities in the Netherlands.” Journal of Policy and Practice in Intellectual Disabilities 21, no. 2: e12496. 10.1111/jppi.12496.

[jir70101-bib-0021] Kerr, H. , K. Widger , G. Cullen‐Dean , J. Price , and P. O'Halloran . 2020. “Transition From Children's to Adult Services for Adolescents/Young Adults With Life‐Limiting Conditions: Developing Realist Programme Theory Through an International Comparison.” BMC Palliative Care 19: 115. 10.1186/s12904-020-00620-2. 32731863 PMC7393825

[jir70101-bib-0022] Klein Haneveld, M. J. , K. Świeczkowska , T. Grybek , et al. 2025. “Transition From Children's to Adults' Healthcare for Youth With (Genetic) Intellectual Disabilities: An ERN‐ITHACA Guideline.” Journal of Intellectual Disability Research 70, no. 1: 29–47. 10.1111/jir.70049. 41115693 PMC12703057

[jir70101-bib-0023] Klein Haneveld, M. J. , K. Vyshka , C. M. W. Gaasterland , et al. 2025. “Mind the Gap’—A Survey on Care Gaps and Priorities for the Transition to Adult Healthcare According to Caregivers of Young People With Rare Conditions Associated With Intellectual Disability.” Journal of Intellectual Disability Research 69, no. 6: 480–488. 10.1111/jir.13229. 40083267 PMC12051228

[jir70101-bib-0024] Kokorelias, K. M. , M. A. M. Gignac , G. Naglie , and J. I. Cameron . 2019. “Towards a Universal Model of Family Centered Care: A Scoping Review.” BMC Health Services Research 19, no. 1: 564. 10.1186/s12913-019-4394-5. 31409347 PMC6693264

[jir70101-bib-0025] Kruithof, K. , E. Olsman , A. Nieuwenhuijse , and D. Willems . 2022. “Parents' Views on Medical Decisions Related to Life and Death for Their Ageing Child With Profound Intellectual and Multiple Disabilities: A Qualitative Study.” Research in Developmental Disabilities 121: 104154. 10.1016/j.ridd.2021.104154. 34954670

[jir70101-bib-0026] Luitwieler, N. , J. Luijkx , C. P. van der Schans , A. A. J. van der Putten , and A. Waninge . 2024. “Experiences and Support Needs of Families Raising Adolescents With Profound Intellectual and Multiple Disabilities During the Transition to Adulthood.” International Journal of Child, Youth, and Family Studies 15, no. 3: 69–100. 10.18357/ijcyfs153202422164.

[jir70101-bib-0027] Matérne, M. , and M. Holmefur . 2022. “Residential Care Staff Are the Key to Quality of Health Care for Adults With Profound Intellectual and Multiple Disabilities in Sweden.” BMC Health Services Research 22, no. 1: 228. 10.1186/s12913-022-07641-y. 35183187 PMC8857860

[jir70101-bib-0028] McManus, M. , P. White , A. Schmidt , et al. 2020. “Health Care Gap Affects 20% of United States Population: Transition From Pediatric to Adult Health Care.” Health Policy OPEN 1: 100007. 10.1016/j.hpopen.2020.100007. 37383315 PMC10297759

[jir70101-bib-0029] Mol‐Bakker, A. , A. A. J. Van Der Putten , W. P. Krijnen , and A. Waninge . 2024. “Physical Health Conditions in Young Children With Profound Intellectual and Multiple Disabilities: The Prevalence and Associations Between These Conditions.” Child: Care, Health and Development 50, no. 2: e13252. 10.1111/cch.13252. 38520205

[jir70101-bib-0030] Nakken, H. , and C. Vlaskamp . 2007. “A Need for a Taxonomy for Profound Intellectual and Multiple Disabilities.” Journal of Policy and Practice in Intellectual Disabilities 4, no. 2: 83–87. 10.1111/j.1741-1130.2007.00104.x.

[jir70101-bib-0031] NICE Guideline . 2023. “Transition From Childrens' to Adults' Services for Young People Using Health or Social Care Services (Updated Version).” https://www.nice.org.uk/guidance/qs140/resources/transition‐from‐childrens‐to‐adults‐services‐pdf‐75545472790213. 10.1136/archdischild-2017-313208 29269436

[jir70101-bib-0032] Parsons, H. M. , H. I. Abdi , V. A. Nelson , et al. 2022. “Transitions of Care From Pediatric to Adult Services for Children With Special Healthcare Needs.” 10.23970/ahrqepccer255. 37184175

[jir70101-bib-0033] Patel, D. R. , R. Apple , S. Kanungo , and A. Akkal . 2018. “Narrative Review of Intellectual Disability: Definitions, Evaluation and Principles of Treatment.” Pediatric Medicine 1: 11. 10.21037/pm.2018.12.02.

[jir70101-bib-0034] Raap, E. , K. L. Weille , and C. Dedding . 2024. “‘It Is Up to Me Because I Gave Him This Life’ How the Awareness of Being Permanently and Unconditionally Responsible Shapes the Experience of Chronic Sorrow in Parents of Disabled Children.” Psychology and Health 40, no. 12: 2055–2075. 10.1080/08870446.2024.2378736. 39129195

[jir70101-bib-0035] Rousseau, M. , I. Hamouda , M. Aim , et al. 2024. “Health Status of Individuals With Polyhandicap Across a 5‐Year Follow‐Up Period.” Scientific Reports 14, no. 1: 23197. 10.1038/s41598-024-74102-3. 39369038 PMC11455907

[jir70101-bib-0036] Schmidt, A. , S. M. Ilango , M. A. McManus , K. K. Rogers , and P. H. White . 2020. “Outcomes of Pediatric to Adult Healthcare Transition Interventions: An Updated Systematic Review.” Journal of Pediatric Nursing 51: 92–107. 10.1016/j.pedn.2020.01.002. 31981969

[jir70101-bib-0037] Sturm, J. , A. Van Staa , J. C. Escher , and J. Sattoe . 2024. “Exploring Six Successful Nurse‐Led Transition Clinics: Experiences and Outcomes.” Health Care Transitions 2: 100071. 10.1016/j.hctj.2024.100071. 39712599 PMC11658268

[jir70101-bib-0038] Trettin, B. , N. Hyltoft , H. Agerskov , C. Nielsen , and C. E. Frandsen . 2025. “From Pediatrics to Adult Care—Experiences of Transition Among Youth With a Chronic Medical Condition: A Meta‐Ethnography.” Health Care Transitions 3: 100118. 10.1016/j.hctj.2025.100118. 40917824 PMC12408251

[jir70101-bib-0039] van der Putten, A. , C. Vlaskamp , J. Luijkx , and P. Poppes . 2017. “Children and Adults With Severe Intellectual and Multiple Disabilities: Time for a New Perspective. Position Paper Research Centre EMB”.

[jir70101-bib-0041] Van Timmeren, E. A. , A. A. J. Van Der Putten , H. M. J. Van Schrojenstein Lantman‐de Valk , C. P. Van Der Schans , and A. Waninge . 2016. “Prevalence of Reported Physical Health Problems in People With Severe or Profound Intellectual and Motor Disabilities: A Cross‐Sectional Study of Medical Records and Care Plans.” Journal of Intellectual Disability Research 60, no. 11: 1109–1118. 10.1111/jir.12298. 27197564

[jir70101-bib-0042] Witkamp, E. , M. Droger , R. Janssens , L. Van Zuylen , and A. Van Der Heide . 2016. “How to Deal With Relatives of Patients Dying in the Hospital? Qualitative Content Analysis of Relatives' Experiences.” Journal of Pain and Symptom Management 52, no. 2: 235–242. 10.1016/j.jpainsymman.2016.02.009. 27090852

[jir70101-bib-0043] Zandbelt, L. M. , E. J. B. Gijssel , C. H. Coppens , et al. 2025. “Health Problems in Children With Profound Intellectual and Multiple Disabilities: A Scoping Review.” European Journal of Pediatrics 184: 67. 10.1007/s00431-024-05876-x. PMC11624250 39641840

