# Supplementary material for: Factors Influencing the Implementation of Transitional Care for Adolescents With Profound Intellectual and Multiple Disabilities: Experiences of Dutch Healthcare Professionals
Source: J Intellect Disabil Res. 2026 Mar 13;70(9):935–44. doi: 10.1111/jir.70101 (PMC13537372; doi:10.1111/jir.70101)
Supplement: Supplementary file 1 [file JIR-70-935-s001.docx]

Supplements

## Supplement 1 Topic list

Table 1 Topic list interviews healthcare professionals

|  | Theme | Questions (example) |
| --- | --- | --- |
| 1 | General information on participant and institution | - At what age do you start discussing the subject of transitional care? |
| 2 | General questions | - When do you consider your transitional care as successful? - What main outcome measure would you choose for transitional care? |
| 3 | Organisation of transitional care | - Is there a designated transitional care coordinator / team in your institution? - Do you use a protocol or step-by-step plan? |
| 4 | Communication with colleagues and families | - How do you transfer patient information to your colleague(s)? - How do you inform families about transitional care? |
| 5 | Experienced enabling and preventing factors | - If there were no restrictions in funding / time / regulations, what would you change to improve transitional care? - What would be a point for improvement for physicians? |

## Supplement 2 Coding list

| Open codes | Levels | Domains by Flottorp et al. |
| --- | --- | --- |
| 1. Degree of complexity in adolescents with PIMD 2. Degree of heterogeneity in adolescents with PIMD 3. Parental role 4. Continuous parental involvement 5. Degree of self-reliance (parents) 6. Emotional burden for parents 7. Administrative burden for parents 8. Parents reluctant to leave PC and transfer to AC 9. Level of shared decision making 10. Period of transition: renewed mourning / coping with loss 11. Parental factors influencing parental possibilities: poverty, cultural background, intellectual disability (in parents), immigrant background, language barrier | (inter)personal level | Patient & family factors |
| 12. HCP’s skills – communication with non-verbal adolescents and their parents 13. HCP’s knowledge – hereditary and congenital disabilities 14. HCP’s attitude: having a holistic view on adolescent and health care in general, like to puzzle since healthcare problems might be atypical / hard to diagnose 15. HCP’s network for referring adolescents from PC to AC 16. In general: PC uses a more holistic view on healthcare whereas AC tends to be more specialty organised 17. HCPs prefer personalized healthcare | (inter)personal level  Organisational level | Individual HCP factors |
| 18. HCPs prefer warm handovers in complex cases 19. Difficulties in information transfer due to HCPs being related to different healthcare facilities (e.g. hospital, residential facility, GP etc) 20. Role of the GP => should be involved, but usually is not in childhood due to high complexity 21. Finding appropriate healthcare follow up is a puzzle 22. The need to put transitional care and its importance on the agenda of policy makers and (higher) healthcare management | (inter)personal level  Organisational level | Professional interactions |
| 23. Most important outcome measure of transitional care: continuity of care 24. Parents should know who to call for questions concerning the healthcare of their child 25. In the event of a medical emergency / urgency, all those involved must know who has which responsibilities and should be able to help the adolescent and the family 26. A medical coordinator in AC should follow up the coordinating role of the paediatrician in PC 27. Healthcare provider in PC should timely start conversation on transitional care to ensure enough time to organise matters | Organisational level | Guideline factors |
| 28. Shortage of ID physicians 29. Shortage of physicians in AC who are familiar with treating adolescents with PIMD 30. Waiting lists for AC leading to delayed transfers to AC and adolescents staying in PC after their 18^th^ birthday 31. IDphysician does not have access to hospital facilities (consultation room, ICT, communication systems, beds in case of hospitalization | Organisational level  Environmental level | Capacity for organisational change |
| 32. Funding warm handovers 33. Funding extended consultation time 34. Funding multidisciplinary consultations 35. Wish for destandardisation of healthcare (practice and funding) due to heterogeneity and complexity of PIMDgroup | Environmental level | Incentives and resources |
| 36. Transitional care involves more than a medical transition 37. HCPs want to inform parents of ‘things to do list’ 38. HCPs cannot help parents with administrative load 39. Need for support for families 40. Information about transitional process is fragmented 41. Some parents choose not to take in information (yet), which means they may miss pivotal moments in transitional care process | Environmental level  (inter)personal level | Social political and legal factors |
